# Independent and joint associations of monocyte to high-density lipoprotein-cholesterol ratio and body mass index with cardiorenal syndrome: insights from NHANES 2003–2020

**DOI:** 10.1186/s12944-024-02149-2

**Published:** 2024-05-23

**Authors:** Junjie Lin, Zixin Li, Jiamin Xu, Mengshan Pan, Tongle Yin, Jiadong Wang, Qinghua Sun, Weijun Zheng, Rucheng Chen

**Affiliations:** 1 School of Public Health, Zhejiang Chinese Medical University, 548 Binwen Road, Binjiang District, Hangzhou City, 310053 Zhejiang Province China; 2 Department of Clinical Medicine, School of Medicine, Hangzhou City University, Hangzhou, 310015 China

**Keywords:** Monocyte to high-density lipoprotein-cholesterol ratio, Body mass index, Cardiorenal syndrome, NHANES, Public Health

## Abstract

**Background:**

With the development of pathophysiology, cardiorenal syndrome (CRS), a complex and severe disease, has received increasing attention. Monocyte to high-density lipoprotein-cholesterol ratio (MHR) and body mass index (BMI) are independent risk factors for cardiovascular diseases, but their association with CRS remains unexplored. This study aims to explore the independent and joint effects of MHR and BMI on CRS.

**Methods:**

We included 42,178 NHANES participants. The determination of CRS referred to the simultaneous presence of cardiovascular disease (identified through self-report) and chronic kidney disease (eGFR < 60 mL/min per 1.73 m²). We employed multivariate weighted logistic regression to evaluate the odds ratio (OR) and 95% confidence interval (CI) for the independent and joint associations of MHR and BMI with CRS. We also conducted restricted cubic spines to explore nonlinear associations.

**Results:**

The prevalence of CRS was 3.45% among all participants. An increase in both MHR and BMI is associated with a higher risk of CRS (MHR: OR = 1.799, 95% CI = 1.520–2.129, *P* < 0.001, *P*-trend < 0.001; BMI: OR = 1.037, 95% CI = 1.023–1.051, *P* < 0.001). Individuals who simultaneously fall into the highest quartile of MHR and have a BMI of 30 or more face the highest risk of CRS compared to those in the lowest MHR quartile with a BMI of less than 25 (OR = 3.45, 95% CI = 2.40–4.98, *P* < 0.001). However, there is no interactive association between MHR and BMI with CRS.

**Conclusions:**

Higher MHR and BMI are associated with higher odds of CRS. MHR and BMI can serve as tools for early prevention and intervention of CRS, respectively.

**Supplementary Information:**

The online version contains supplementary material available at 10.1186/s12944-024-02149-2.

## Introduction

Cardiorenal Syndrome (CRS) involves complex interactions between cardiovascular and renal systems, classified into five types by The Acute Dialysis Quality Initiative in 2009 [1], including CRS induced by acute or chronic cardiovascular function decline, CRS caused by acute or chronic renal function deterioration, and CRS secondary to other physical conditions [1]. CRS exhibits a high prevalence, particularly in patients with heart failure, where it exceeds 50%, presenting significant management challenges for public health and healthcare systems [2]. Recently, renewed focus has been placed on CRS’s pathophysiology, diagnosis, and treatment strategies, highlighting significant gaps in our understanding of its fundamental mechanisms, risk factors, and predictors [3]. Therefore, it is increasingly important to identify potential risk factors and biomarkers for CRS through epidemiological studies, which could significantly enhance the prevention and management of CRS [3].

High-density lipoprotein-cholesterol (HDL-C) has been acknowledged for its significant role as a cardiovascular protector, owing to its potent antioxidative capabilities and crucial function in maintaining and enhancing vascular elasticity [4, 5]. Furthermore, the reduction in plasma HDL-C levels appears to be only lipid alteration definitively associated with the progression of chronic kidney disease (CKD) [6]. However, the role of monocytes is quite the opposite; they can induce local ischemia and inflammation, thereby promoting arteriosclerosis, a key risk factor for CKD and cardiovascular diseases (CVD) [7–9]. As a relatively new biomarker, the Monocyte to High-Density Lipoprotein-Cholesterol Ratio (MHR) combines two independent CVD risk factors, surpassing the predictive ability of monocyte count alone for CVD and reflecting the balance between chronic inflammation and oxidative stress [10]. Past epidemiological studies have extensively explored the association between elevated MHR and adverse cardiovascular events, demonstrating efficient predictive power for all-cause and cardiovascular mortality [11–14]. However, research on its correlation with CKD and CRS is quite limited.

Body mass index (BMI), widely used as an indicator of obesity, is recognized as a risk factor for both CVD and kidney ailments [15, 16]. However, BMI fails to encompass various metabolic markers, such as adipokine and inflammatory markers, which often play pivotal roles in the onset and progression of CVD and CKD [15, 16]. Furthermore, BMI, monocytes, and HDL interact complexly, and individuals with high BMI may also exacerbate cardiorenal syndrome due to complications from adipose tissue dysfunction [16–19].

Current research on biomarkers for CRS primarily focuses on prognosis and is limited by sparse clinical data. There is a significant scarcity of large-scale epidemiological studies that could delve into the disease’s mechanisms to inform prevention and treatment strategies [20]. Therefore, this study utilizes data from the National Health and Nutrition Examination Survey (NHANES) to explore the independent, combined, and potential interactive associations of MHR and BMI with CRS. It aims to fill research gaps in CRS risk factors and predictors, offering more personalized and effective prevention strategies.

## Materials and methods

### Data and study participants

We conducted a cross-sectional study using data from NHANES 2003 to 2020, a program overseen by the National Center for Health Statistics designed to assess adults’ and children’s health and nutritional status in the United States. The NHANES includes interviews covering demographic, socioeconomic, dietary, and health-related aspects, along with examinations involving various medical tests and evaluations of blood markers by trained medical personnel.

**Fig. 1:**
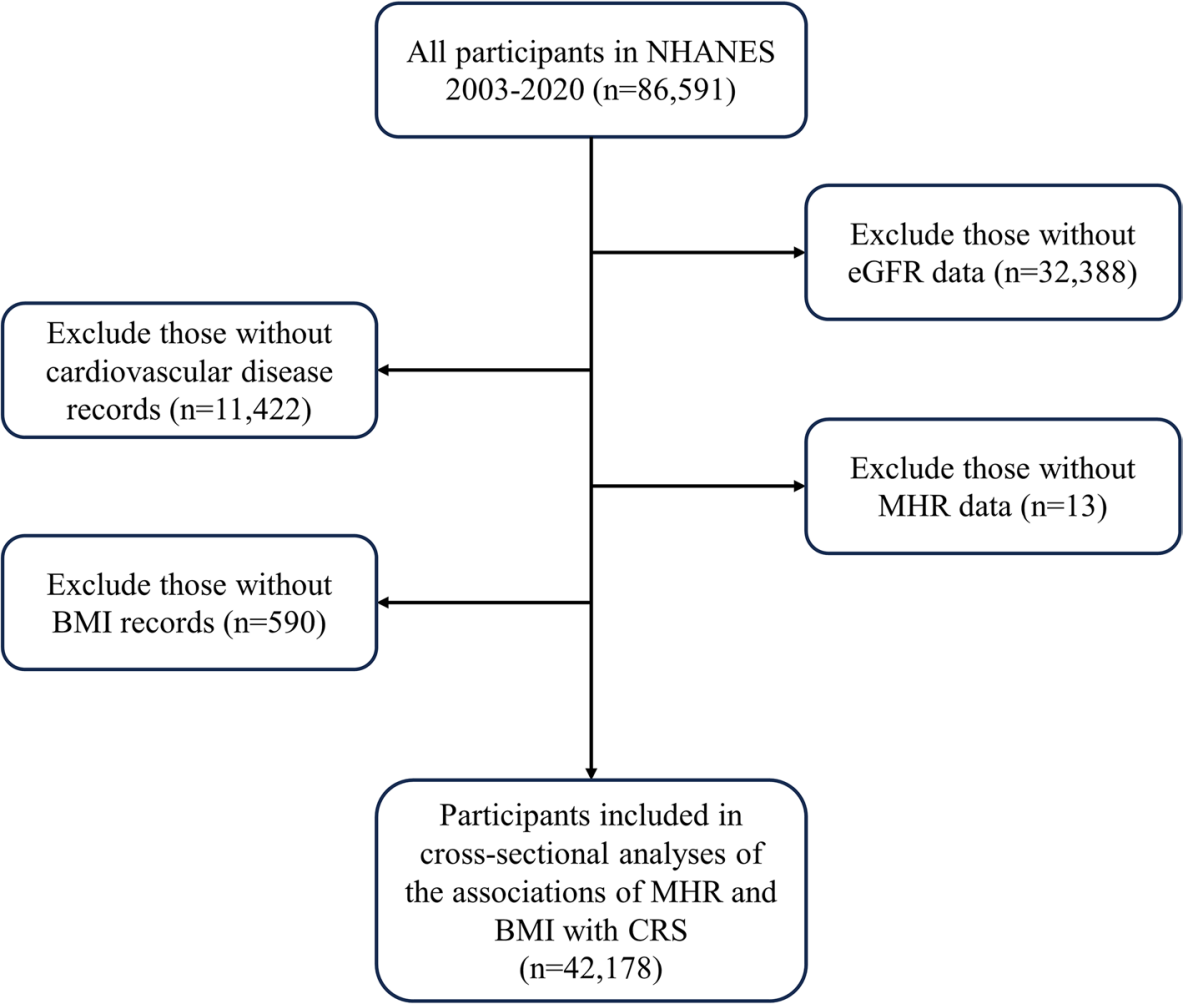
Inclusion and exclusion process of the study participants. Abbreviations: NHANES, National Health and Nutrition Examination Survey; eGFR, estimated glomerular filtration rate; MHR, high-density lipoprotein-cholesterol ratio; CRS, cardiorenal syndrome

Eighty-six thousand five hundred ninety-one individuals participated in the eight rounds of the NHANES survey from 2003 to 2020. We excluded 32,388 individuals for whom CKD assessment was not possible due to the lack of necessary data to calculate the estimated glomerular filtration rate (eGFR), 11,142 participants who lacked CVD information, 13 individuals missing MHR values, and 590 participants without BMI records. Figure 1 demonstrates the specific process of selecting the participants.

### Assessment of high-density lipoprotein-cholesterol ratio and body Mass Index

The formula for calculating MHR is *MHR = Monocyte count (×10*^*3*^*cells/ul) / High-density Lipoprotein-cholesterol (mg/dL)*. Blood specimens were collected in the morning after at least nine hours of overnight fasting. For measuring monocytes, use three simultaneous measurements of laser light scatter, high-frequency conductivity, and individual cell volume on the Beckman Coulter MAXM, and for determining the concentration of HDL-C, use precipitation or immunoassay on Roche Cobas 6000.

The formula for calculating BMI is *BMI = Weight in kilograms / Height in meters squared*. Physical measurement data were collected by trained health technicians at the Mobile Examination Centers and reviewed for anomalies.

### Cardiorenal syndrome ascertainment

Referring to the previous study on NHANES participants, we defined CRS as an individual simultaneously suffering from CVD and CKD [21]. CVD was determined by the self-reported diagnosis of coronary disease, angina, congestive heart, heart attack, or stroke [21]. We determined CKD by assessing the eGFR, with eGFR values below 60 mL/min per 1.73 m² indicating CKD. We utilized the CKD-Epidemiology Collaboration (EPI) equation to calculate *eGFR*: $eGFR=141\times {{min}\left(\frac{Scr}{k},1\right)}^{\alpha }\times {{max}\left(\frac{Scr}{k},1\right)}^{-1.209}\times {0.993}^{age}\times 1.018\left[if female\right]\times 1.159\left[if black\right]$, where Scr denotes serum creatinine concentration (mg/dL) measured by the Jaffe rate methods, *k* is 0.9 for males and 0.7 for females, and α is -0.411 for males and − 0.329 for females [22].

### Covariates

We included sociodemographic characteristics, health-related behaviors, and some confounding factors as covariates. The sociodemographic characteristics comprise age, gender, race, marital status, and education level. Health-related behaviors include smoking and drinking status. Confounding factors encompass hypertension and diabetes. All other variables are categorical except age, with specific categorization methods detailed in Supplementary Table 1.

### Statistical analysis

In descriptive statistical analysis, we categorized participants into non-CRS and CRS groups. We used mean ± standard deviation (SD) to describe continuous variables and compared them between the two groups using the Student’s t-test. For categorical variables, we described them using frequency and percentage and compared them using the Chi-square test.

NHANES employs a complex multistage probability sampling design. Following the NHANES analytical guidelines, we considered sampling weights to obtain national estimates that reflect the true relative proportions of different subgroups in the overall population of the United States. We conducted multiple imputations for missing values in covariates. Supplementary Table 2 shows details of the proportion of missing data and the multiple imputation. We used weighted multivariate Logistic regression with three models to estimate odds ratios (ORs) and their 95% confidence interval (95% CIs) of independent associations of MHR and BMI with CRS. Model 1 adjusted for demographic characteristics, including age, gender, race, marital status, and education. Model 2, building upon Model 1, further adjusted for health-related behaviors, including smoking and drinking status. Model 3 extended these adjustments to include hypertension and diabetes. Considering that MHR is a skewed distribution and its values are relatively small, which might lead to less intuitive effect sizes, we performed a logarithmic transformation using the natural logarithm. Supplementary Fig. 1 provides the distribution of MHR and the natural logarithmic transformation of MHR (Ln MHR). We also categorized the MHR into four levels based on quartiles, using the first quartile as the reference group for trend tests. Furthermore, we employed three-knot restricted cubic spines (RCS) to explore the possibility of non-linear associations of MHR and BMI with CRS.

To evaluate the joint associations of MHR and BMI with CRS, we stratified participants into twelve groups based on MHR (4 quartiles) and BMI (3 groups: <25 kg/m^2^, 25–30 kg/m^2^, and ≥ 30 kg/m^2^). Taking individuals with BMI < 25 and MHR in the first quartile as the control group, we assessed the OR values and 95% CI for CRS in each group. Additionally, we conducted stratified analyses on the association between MHR (including continuous and categorical variables) and CRS based on three subgroups of BMI. The interaction effects were determined by performing an ANOVA analysis comparing regression models with and without interaction terms [23]. We also conducted several sensitivity analyses to assess the robustness of our findings as follows: 1). analyzing the data before multiple imputation to explore the potential impact that multiple imputation might have on the results; 2). without considering weights to mitigate the potential over-adjustment bias associated with including main variables as covariates after calculating sampling weights; 3). conducting the Poisson regression model.

All analyses were performed using R Studio (Version 4.3.2) and Storm Statistical Platform (www.medsta.cn/software). We considered a two-sided *P* value < 0.05 to be significant.

## Results

### Baseline characteristics

In the NHANES 2003–2020 cycles, 42,178 subjects met the criteria for this cross-sectional study. Supplementary Table 3 compares the baseline characteristics of the included and excluded subjects. Most of the excluded subjects were minors who were not asked about relevant diseases or were not applicable for indicator measurements.

Among the included subjects, the mean (SD) age of those without CRS was 48.69 (17.91), while 73.89 (17.79) for those with CRS. The MHR of CRS patients was significantly higher than that of non-CRS participants, and the proportion of males, as well as those with hypertension and diabetes, was significantly higher in CRS patients compared to non-CRS participants. Table 1 displays the characteristics of participants according to CRS, and Supplementary Tables 4 and 5 show the characteristics of participants according to MHR and BMI. Supplementary Table 6 provides the eGFR levels of patients with cardiorenal syndrome and the baseline characteristics of participants according to eGFR.

**Table 1:** Baseline characteristics of participants according to CRS

Variables^a^	Without CRS	With CRS	t/χ^2^	*P*
**Age**	48.69 ± 17.53	73.89 ± 8.73	105.14	< 0.001
**Gender**			18.32	< 0.001
Male	19,647 (48.2%)	785 (54.0%)		
Female	21,076 (51.8%)	670 (46.0%)		
**Race**			318.71	< 0.001
Mexican American	6,621 (16.3%)	89 (6.1%)		
Other Hispanic	3,791 (9.3%)	75 (5.2%)		
Non-Hispanic White	17,236 (42.3%)	931 (63.9%)		
Non-Hispanic Black	8,654 (21.3%)	289 (19.9%)		
Other Race	4,421 (10.8%)	71 (4.9%)		
**Marriage status**			399.63	< 0.001
Married/Living with a partner	24,603 (60.4%)	721 (49.6%)		
Widowed/Divorce/Separated	10,077 (24.8%)	675 (46.4%)		
Never married	6,023 (14.8%)	58 (4.0%)		
**Education level**			90.23	< 0.001
High school or below	19,290 (47.4%)	873 (60.1%)		
University or above	21,394 (52.6%)	580 (39.9%)		
**Smoking**			366.07	< 0.001
Never	22,659 (55.6%)	627 (43.1%)		
Smoking cessation	9,614 (23.7%)	658 (45.2%)		
Smoking at present	8,450 (20.7%)	170 (11.7%)		
**Drinking**			500.24	< 0.001
No	6,897 (21.2%)	550 (49.6%)		
Yes	25,595 (78.8%)	559 (50.4%)		
**Hypertension**			1205.77	< 0.001
No	24,219 (59.5%)	200 (13.7%)		
Yes	16,495 (40.5%)	1,255 (86.3%)		
**Diabetes**			888.66	< 0.001
No	34,782 (85.4%)	823 (56.6%)		
Yes	6,941 (14.6%)	632 (43.4%)		
**BMI (kg/m** ^**2**^ **)**			34.03	< 0.001
< 25	11,770 (28.9%)	326 (22.4%)		
25–30	13,562 (33.3%)	492 (33.8%)		
≥ 30	15,391 (37.8%)	637 (43.8%)		
**MHR**	0.011 ± 0.0063	0.014 ± 0.0080	12.98	< 0.001
**Monocyte count (×10** ^**3**^ **cells/µl)**	0.56 ± 0.203	0.64 ± 0.249	12.27	< 0.001
**HDL-C (mg/dL)**	53.51 ± 16.226	49.80 ± 15.562	8.58	< 0.001

^a^Continuous variables are represented by Mean±SD, while categorical variables are denoted by (n, %)

### Independent and joint associations of MHR and BMI with CRS

**Fig. 2:**
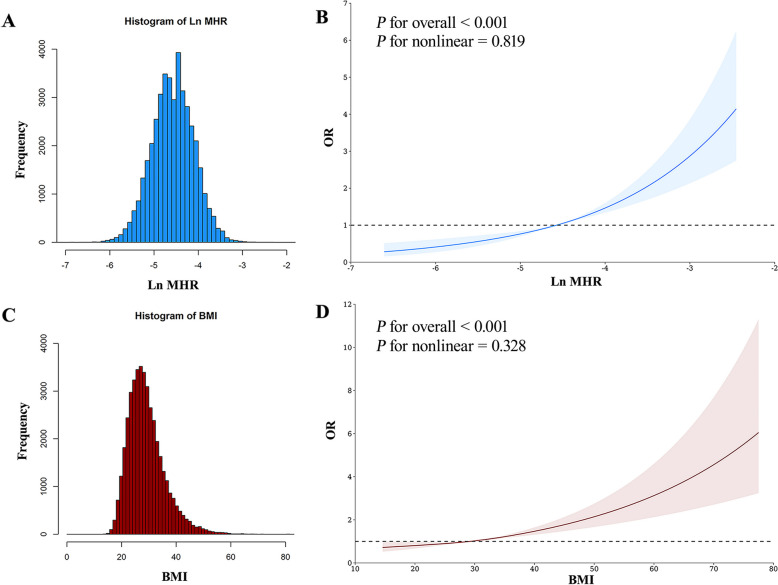
Nonlinear associations of Ln MHR and BMI with CRS. (**A**) Distribution for Ln MHR. (**B**) Restricted cubic spline for the association between Ln MHR and CRS. (**C**) Distribution for BMI. (**D**) Restricted cubic spline for the association between BMI and CRS. Abbreviation: OR, odds ratio; MHR, monocyte to high-density lipoprotein-cholesterol ratio; BMI, body mass index

**Fig. 3:**
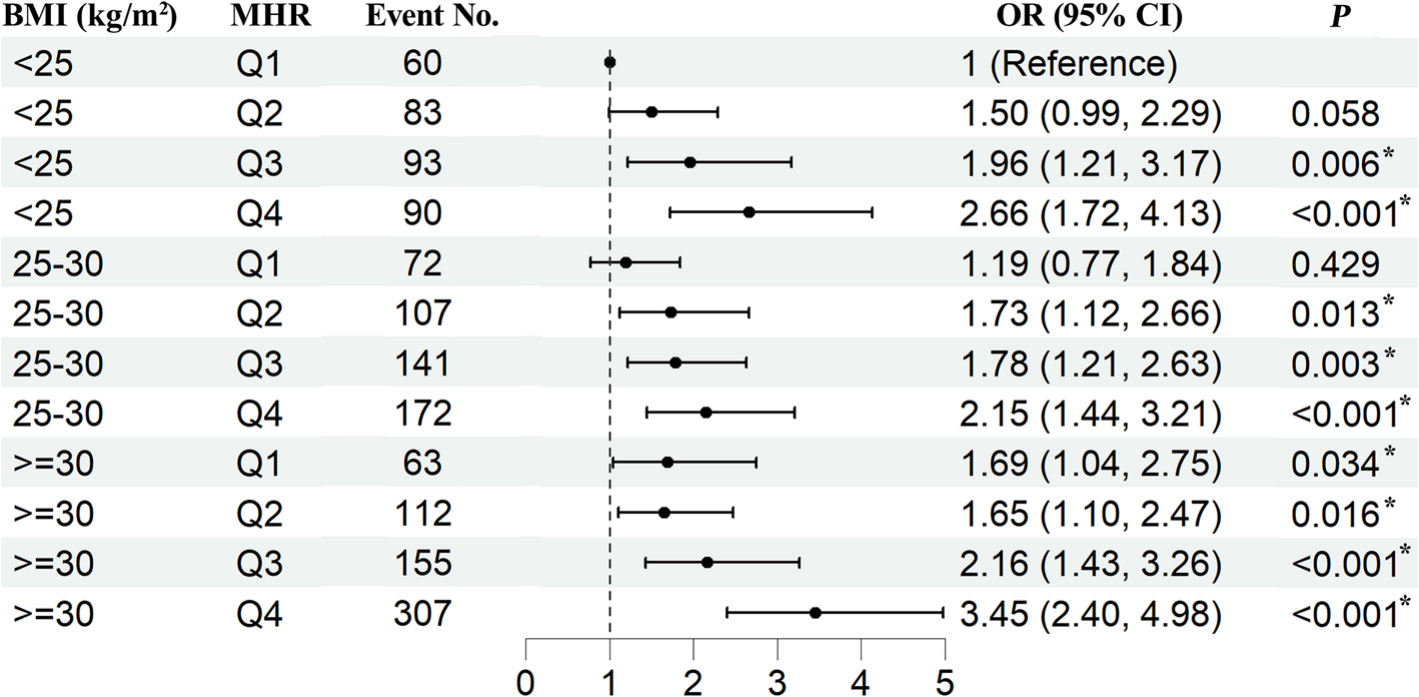
Joint associations of MHR and BMI with CRS in a fully adjusted model. Abbreviation: OR, odds ratio; MHR, monocyte to high-density lipoprotein-cholesterol ratio; BMI, body mass index; CI, confidence interval

Among the 42,178 participants included in the study, the prevalence of CRS was 3.45%. Supplementary Table 7 provides the results of the univariate analysis. After adjusting covariates, weighted Logistic regression suggests an increase in the MHR is associated with a higher prevalence odds of CRS (Ln MHR: OR = 1.799, 95% CI = 1.520–2.129, *P* < 0.001, Table 2). Compared to the first quartile of MHR, each increase in a quartile of MHR is significantly associated with an increased OR for CRS, and there is a significant trend (*P*-trend < 0.001, Table 2). Similarly, each increase of 1 in BMI is associated with higher odds of CRS (OR = 1.037, 95% CI = 1.023–1.051, *P* < 0.001, Table 3). Compared to participants with a BMI of less than 25, those with a BMI of 30 or higher have a higher prevalence of CRS (OR = 1.453, 95% CI = 1.177–1.794, *P* < 0.001, Table 3). Figure 2 displays the distribution of BMI and Ln MHR and reveals no nonlinear association of BMI and Ln MHR with CRS through restricted cubic spline analysis. We further explore the joint association of Ln MHR and BMI with CRS. Figure 3 shows that the OR for individuals of BMI ≥ 30 kg/m^2^ and quartile 4 of Ln MHR compared with those with BMI < 25 kg/m^2^ and quartile 1 of Ln MHR was 3.45 (95% CI = 2.40–4.98, *P* < 0.001).

**Table 2:** Cross-sectional association between Ln MHR and CRS

Subgroup	Ln MHR	Prevalence	Model 1*			Model 2**			Model 3***		
			OR	95% CI	*P*	OR	95% CI	*P*	OR	95% CI	*P*
**Overall**	-4.57 ± 0.48	3.45%	2.334	1.981–2.749	< 0.001	2.223	1.891–2.615	< 0.001	1.799	1.520–2.129	< 0.001
**Quartile 1**	-5.18 ± 0.25	1.85%		Ref.			Ref.			Ref.	
**Quartile 2**	-4.72 ± 0.09	2.84%	1.464	1.146–1.872	0.002	1.427	1.111–1.828	0.005	1.313	1.021–1.690	0.034
**Quartile 3**	-4.41 ± 0.09	3.56%	1.984	1.554–2.558	< 0.001	1.921	1.501–2.457	< 0.001	1.585	1.229–2.043	< 0.001
**Quartile 4**	-3.96 ± 0.24	5.63%	3.157	2.498–3.990	< 0.001	2.963	2.349–3.739	< 0.001	2.275	1.793–2.887	< 0.001
***P*** **-trend**				< 0.001			< 0.001			< 0.001	

* Model 1: Adjusted for age, gender, race, marital status, and education

** Model 2: Additional adjusted smoking status and drinking status

*** Model 3: Additional adjusted hypertension and diabetes

**Table 3:** Cross-sectional association between BMI and CRS

Subgroup	BMI	Prevalence	Model 1*			Model 2**			Model 3***		
			OR	95% CI	*P*	OR	95% CI	*P*	OR	95% CI	*P*
**Overall**	29.19 ± 6.94	3.45%	1.057	1.044–1.070	< 0.001	1.058	1.046–1.071	< 0.001	1.037	1.023–1.051	< 0.001
**< 25 kg/m** ^**2**^	22.20 ± 2.00	2.69%		Ref.			Ref.			Ref.	
**25–30 kg/m** ^**2**^	27.41 ± 1.42	3.50%	1.170	0.956–1.432	0.127	1.196	0.978–1.462	0.080	1.047	0.851–1.288	0.664
**≥ 30 kg/m** ^**2**^	36.02 ± 5.96	3.97%	1.993	1.628–2.440	< 0.001	2.028	1.661–2.475	< 0.001	1.453	1.177–1.794	< 0.001

* Model 1: Adjusted for age, gender, race, marital status, and education

** Model 2: Additional adjusted smoking status and drinking status

*** Model 3: Additional adjusted hypertension and diabetes

### Interactive effect and sensitivity analyses

**Fig. 4:**
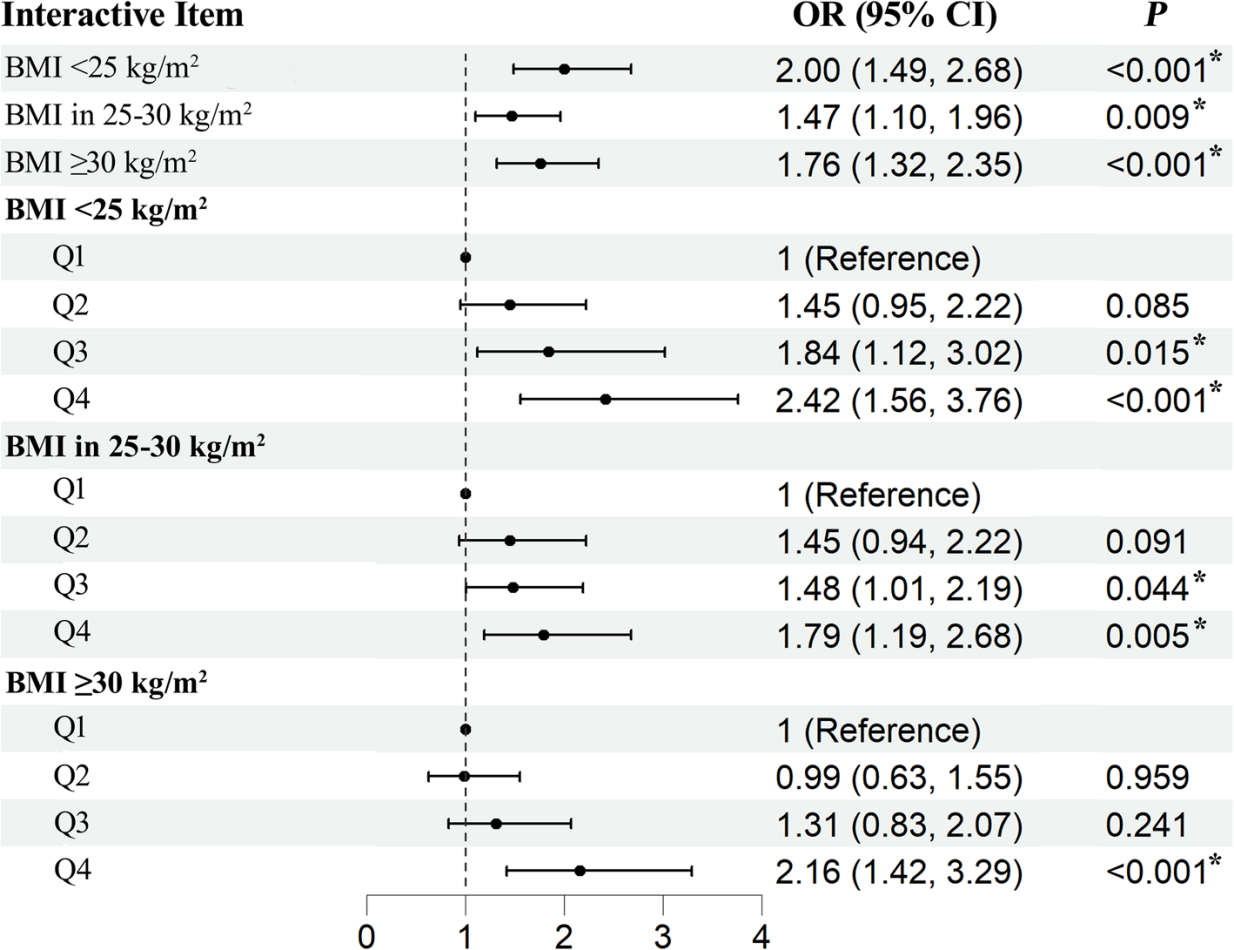
Associations of MHR with CRS by BMI in a fully adjusted model. Abbreviation: OR, odds ratio; MHR, monocyte to high-density lipoprotein-cholesterol ratio; BMI, body mass index; CI, confidence interval

Figure 4 shows the associations of MHR with CRS by BMI, and no significant interactive effect was found between MHR and BMI on CRS (*P*-interaction for the continuous form of MHR is 0.248, and for the quartile form of MHR is 0.363). Supplementary Tables 8–12 present the results of sensitivity analyses, showing that imputation and weighted analysis do not affect the interpretation of the results and that the independent and joint associations of MHR and BMI with CRS remain robust in the Poisson regression model.

## Discussion

In this national cross-sectional study, we revealed that increased MHR and BMI are associated with a higher prevalence odds of CRS without a nonlinear trend. Moreover, individuals who simultaneously fall into the top quartile of MHR and have a BMI of 30 or more have the highest prevalence odds of CRS. Additionally, we did not find any interactive effect in the association of MHR and BMI with CRS.

### Comparison with other studies and mechanisms

Previous epidemiological studies based on Chinese populations have explored various risk factors for adverse cardiovascular events, particularly acute ischemic stroke [11, 24]. Additionally, a nationwide cohort study in the United States revealed the predictive value of MHR for long-term cardiovascular outcomes in the general population, and a follow-up study from Turkey also demonstrated the predictive capability of MHR for long-term outcomes in patients with acute coronary syndrome [14, 25]. Research on the association between MHR and kidney diseases is limited; only one recent large-scale cross-sectional study in a Chinese sample revealed an independent correlation between elevated MHR levels and CKD [26]. A small-scale cohort study from Turkey found that CKD patients with MHR levels in the highest tertile had a higher risk of concurrent cardiovascular events [27]. The research on the association between obesity, or BMI, and adverse physical conditions is extensive and widely accepted. Previous traditional epidemiological studies and Mendelian randomization studies have both demonstrated the association between obesity, or elevated BMI, and the occurrence of various subtypes of CVDs as well as decline in renal function [28–32].

Two systematic reviews elaborated on the complex interactions between the heart and kidneys, where increased cytokine production induced by oxidative stress, an imbalance between nitric oxide and reactive oxygen species, mitochondrial dysfunction, endothelial dysfunction, and subsequent arteriosclerosis are common risk factors for both organs [3, 33]. The individual and interactive roles of monocytes and high-density lipoproteins in inflammation and arteriosclerosis enable the MHR to predict CRS effectively. For example, CKD is characterized by an expansion of a unique pro-inflammatory intermediate monocyte subtype and an increase in monocyte adhesion to endothelial cells [9]. An elevated monocyte count typically signifies an inflammatory state. They participate in both the onset and the resolution phases of inflammation. During the inflammatory process, monocytes can differentiate into tissue macrophages that significantly enhance the inflammatory response and secrete cytokines that help combat infection and facilitate the resolution of inflammation [34]. HDL-C, commonly known as “good cholesterol,” helps reduce inflammation in the body through mechanisms such as transporting cholesterol away from the arteries and can inhibit the natural oxidation of low-density lipoprotein [35, 36]. Furthermore, an interactive relationship exists between monocytes and HDL-C, where HDL-C can inhibit the expression of endothelial adhesion molecules, thereby reducing monocyte aggregation [17]. An epidemiological study also revealed that considering monocytes and HDL-C concurrently can better predict coronary arteriosclerosis [7]. Additionally, as an independent risk factor for CVD and CKD, obesity is closely linked with both monocytes and HDL-C: In populations with higher BMI, the frequency and cell count of non-classical monocyte subgroups are noticeably higher [18]; an increase in BMI alters lipid-lipoprotein metabolism, leading to elevated plasma total cholesterol and low-density lipoprotein levels, as well as reduced HDL levels, thereby further accelerating the development of arteriosclerosis and increasing the risk of CVD and CKD [16].

### Implications

With the advancement in pathophysiology, the mysterious aspects of CRS, a comorbidity pattern marked by mutual influences of cardiovascular and renal diseases, are gradually being unraveled. However, its high prevalence, complex diagnostic and prognostic markers, challenging treatment strategies, and poor prognosis continue to pose significant challenges for patients, medical institutions, and public health systems [3]. In populations with chronic renal insufficiency, the prevalence of atrial fibrillation reaches 18%, and in patients with acute heart failure, those with declining renal function account for as much as 91% [33, 37]. In recent years, an increasing number of biomarkers have provided valuable information in indicating early cardiovascular and renal damage, predicting CRS, differentiating CRS subtypes, and targeting therapeutic interventions for CRS. These include B-type natriuretic peptide, serum creatinine, albuminuria, neutrophil gelatinase-associated lipocalin, and so on [3]. However, the role of these biomarkers in diagnosing renal diseases in the context of heart failure requires further evaluation [33]. Although different types of CRS may have distinct pathophysiological mechanisms, renal failure is an ominous sign, indicating a need for more complex treatments and generally poorer prognosis [38]. As with other diseases, preventing CRS is more effective than any treatment [39].

Previous reviews and epidemiological studies have indicated that the MHR is a convenient marker for predicting the progression of atherosclerosis, a hallmark of cardiovascular events and CKD, rather than merely using individual monocyte counts or HDL-C levels alone [26, 40]. Our study findings support the association between MHR, an index calculable from routine blood test data, and CRS. Additionally, our study found that individuals experiencing both obesity and high MHR have a higher prevalence of CRS. The global prevalence of obesity continues to evolve dramatically, with an increasing number of CVD and CKD cases attributed to obesity [15, 16, 41]. However, obesity is an easily modifiable risk factor. Previous studies have shown that various weight loss interventions can significantly reduce the risk of adverse cardiovascular events and renal failure [42, 43]. The MHR, a precise, reproducible, and easily measured biomarker, facilitates various aspects of CRS treatment. It assists in preventing CRS during routine examinations, indicates the risk of complications in patients with CVD or CKD, and aids clinicians in timely and accurately selecting specific treatments. Additionally, paying attention to BMI levels may provide more precise and personalized strategies for preventing CRS.

### Strengths and limitations

Our study boasts several advantages. Firstly, it is the pioneering research utilizing national-level data to investigate the relationship between MHR and BMI with CRS. The simultaneous consideration of the individual and combined associations of MHR and BMI holds greater practical significance. Additionally, a complex multistage probability sampling method ensures the representativeness and generalizability of our findings. However, there are also some limitations to the present study. Firstly, self-reported cardiovascular events may be subject to reporting bias. Secondly, while CRS is classified into five subtypes, our study sample only represents chronic CRS, and we cannot determine the sequence of cardiovascular and renal functional decline. Finally, although our study is large-scale, the cross-sectional design does not allow for the establishment of causality. Therefore, future cohort studies are needed to determine the longitudinal association between MHR and other biomarkers with CRS, focusing on whether CRS is secondary to renal function decline or CVD. Additionally, studies based on clinical data are essential to provide insights into the predictiveness and practicality of MHR.

## Conclusions

In summary, our study supports that high MHR and obesity are independent risk factors for CRS, and individuals with both high MHR and obesity have a higher prevalence of CRS. MHR and BMI, as cost-effective indicators, will play a significant role in public health services focused on weight reduction and early prevention efforts for CRS.

### Supplementary Information

Supplementary Material 1.

## Data Availability

No datasets were generated or analysed during the current study.

## Abbreviations

CRS: Cardiorenal Syndrome
MHR: Monocyte to High-density Lipoprotein-cholesterol Ratio
HDL-C: High-Density Lipoprotein-Cholesterol
CKD: Choric Kidney Disease
CVD: Cardiovascular Disease
BMI: Body Mass Index
NHANES: National Health and Nutrition Examination Survey
eGFR: Estimated Glomerular Filtration Rate
SD: Standard Deviation
OR: Odds Ratio
CI: Confidence Interval
Ln MHR: The Natural Logarithmic Transformation of MHR
RCS: Restricted cubic spines
