# Supplementary material for: Independent and joint associations of monocyte to high-density lipoprotein-cholesterol ratio and body mass index with cardiorenal syndrome: insights from NHANES 2003–2020
Source: Lipids Health Dis. 2024 May 23;23:153. doi: 10.1186/s12944-024-02149-2 (PMC11112806; doi:10.1186/s12944-024-02149-2)
Supplement: Supplementary file 1 — Supplementary Material 1. [file 12944_2024_2149_MOESM1_ESM.docx]

**Supplementary Material**

**Supplementary Table 1.** Assessment of covariates

**Supplementary Table 2.** Missing variable analysis

**Supplementary Table 3.** Differences in baseline characteristics between excluded participants and those included

**Supplementary Table 4.** Baseline characteristics of participants according to MHR

**Supplementary Table 5.** Baseline characteristics of participants according to BMI

**Supplementary Table 6.** Baseline characteristics of participants according to eGFR

**Supplementary Table 7.** Univariate logistic regression

**Supplementary Table 8.** Sensitivity analyses: the association between Ln MHR and CRS

**Supplementary Table 9.** Sensitivity analyses: the association between BMI and CRS

**Supplementary Table 10.** Sensitivity analyses (without multiple imputation): joint associations of MHR and BMI with CRS

**Supplementary Table 11.** Sensitivity analyses (not considering weights): joint associations of MHR and BMI with CRS

**Supplementary Table 12.** Sensitivity analyses (Poisson regression model): joint associations of MHR and BMI with CRS

**Supplementary Figure 1.** Histograms of MHR and Ln MHR

**Supplementary Table 1.** Assessment of covariates

| **Items** | **NHANES (2003-2020)** |
| --- | --- |
| **Age** | ≥18 |
| **Gender** | Male |
|  | Female |
| **Race** | Mexican American |
|  | Other Hispanic |
|  | Non-Hispanic White |
|  | Non-Hispanic Black |
|  | Other Race - Including Multi-Racial |
| **Marriage Status** | Married/Living with a partner |
|  | Widowed/Divorce/Separated |
|  | Never married |
| **Education level** | High school or below |
|  | University or above |
| **Smoking** | Never |
|  | Smoking cessation |
|  | Smoking at present |
| **Drinking** | Yes |
|  | No |
| **Hypertension** | Yes  1. SBP ≥140 mm Hg or  2. DBP ≥90 mm Hg or 3. “Have you ever been told by a doctor or other health professional that you had hypertension”  4. Taking prescriptions for hypertension |
|  | No |
| **Diabetes** | Yes  1. “Have you ever been told by a doctor or other health professional that you had hypertension”  2. Taking insulin now  3. Hemoglobin A1C≥6.5% |
|  | No |

**Supplementary Table 2.** Missing variable analysis*****

| **Variables** | **Missing individuals, n (%)** |
| --- | --- |
| Education | 41 (0.10%) |
| Marital status | 21 (0.05%) |
| Drinking status | 8557 (20.28%) |
| Hypertension | 9 (0.02%) |

* The multiple imputation method used in this study is based on multivariate linear and multivariate logistic regression models. It employs the Monte Carlo simulation method to infer and fill in missing values using observed values from the existing data. Five complete datasets were eventually generated, and by analyzing these complete datasets and combining the results, the outcomes were obtained.

**Supplementary Table 3.** Differences in baseline characteristics between excluded participants and those included

| **Variables*** | **Respondents** | **Included participants**  **(n=42,178)** | **Excluded participants**  **(n=44,413)** | **t/χ2** | ***P*** |
| --- | --- | --- | --- | --- | --- |
| **Age** | 86,591 | 49.56±17.91 | 14.66±17.79 | 287.53 | <0.001 |
| **Gender** |  |  |  | 34.155 | <0.001 |
| Male | 42,829 (49.5%) | 20,432 (48.4%) | 22,397 (50.4%) |  |  |
| Female | 43,762 (50.5%) | 21,746 (51.6%) | 22,016 (49.6%) |  |  |
| **Race** |  |  |  | 1795.43 | <0.001 |
| Mexican American | 16,897 (19.5%) | 6,710 (15.8%) | 10,187 (22.9%) |  |  |
| Other Hispanic | 7,908 (9.1%) | 3,866 (9.2%) | 4,042 (9.1%) |  |  |
| Non-Hispanic White | 31,572 (36.5%) | 18,167 (43.1%) | 13,405 (30.2%) |  |  |
| Non-Hispanic Black | 20,715 (23.9%) | 8,943 (21.2%) | 11,772 (26.5%) |  |  |
| Other Race | 9,499 (11.0%) | 4,492 (10.7%) | 5,007 (11.3%) |  |  |
| **Marriage status** |  |  |  | 4463.39 | <0.001 |
| Married/Living with a partner | 25,544 (55.3%) | 25,324 (60.1%) | 3,329 (34.4%) |  |  |
| Widowed/Divorce/Separated | 12,842 (24.7%) | 10,752 (25.5%) | 2,090 (21.5%) |  |  |
| Never married | 10,353 (20.0%) | 6,081 (14.4%) | 4,272 (44.1%) |  |  |
| **Education level** |  |  |  | 11727.32 | <0.001 |
| High school or below | 43,395 (63.6%) | 20,163 (47.9%) | 23,232 (88.9%) |  |  |
| University or above | 24,879 (36.4%) | 21,974 (52.1%) | 2,905 (11.1%) |  |  |
| **Smoking** |  |  |  | 3963.91 | <0.001 |
| Never | 37,218 (63.1%) | 23,286 (55.2%) | 13,932 (82.9%) |  |  |
| Smoking cessation | 11,757 (19.9%) | 10,272 (24.4%) | 1,485 (8.8%) |  |  |
| Smoking at present | 10,007 (17.0%) | 8,620 (20.4%) | 1,387 (8.3%) |  |  |
| **Drinking** |  |  |  | 3.866 | 0.049 |
| No | 8,218 (22.3%) | 7,447 (22.2%) | 771 (23.7%) |  |  |
| Yes | 28,641 (77.7%) | 26,154 (77.8%) | 2,487 (76.3%) |  |  |
| **Hypertension** |  |  |  | 6606.89 | <0.001 |
| No | 46,549 (69.1%) | 24,419 (57.9%) | 22,130 (87.8%) |  |  |
| Yes | 20,821 (30.9%) | 17,750 (42.1%) | 3,071 (12.2%) |  |  |
| **Diabetes** |  |  |  | 3999.79 | <0.001 |
| No | 75,186 (90.7%) | 35,605 (84.4%) | 39,581 (97.2%) |  |  |
| Yes | 7,720 (9.3%) | 6,573 (15.6%) | 1,147 (2.8%) |  |  |

* Continuous variables are represented by Mean±SD, while categorical variables are denoted by (n, %).

**Supplementary Table 4.** Baseline characteristics of participants according to MHR

| **Variables*** | **Respondents** | **MHR** | **t/F** | ***P*** |
| --- | --- | --- | --- | --- |
| **Total** | 42,178 | 0.01160±0.00635 |  |  |
| **Age** | 49.56±17.91 |  |  |  |
| **Gender** |  |  | 53.53 | <0.001 |
| Male | 20,432 (48.44%) | 0.01323±0.00710 |  |  |
| Female | 21,746 (51.56%) | 0.00999±0.00506 |  |  |
| **Race** |  |  | 170.03 | <0.001 |
| Mexican American | 6,710 (18.55%) | 0.01184±0.00559 |  |  |
| Other Hispanic | 3,866 (10.68%) | 0.01188±0.00569 |  |  |
| Non-Hispanic White | 12,167 (33.62%) | 0.01222±0.00720 |  |  |
| Non-Hispanic Black | 8,943 (24.73%) | 0.01022±0.00531 |  |  |
| Other Race | 4,492 (12.42%) | 0.01085±0.00569 |  |  |
| **Marriage status** |  |  | 18.49 | <0.001 |
| Married/Living with a partner | 25,324 (60.08%) | 0.01170±0.00657 |  |  |
| Widowed/Divorce/Separated | 10,752 (25.51%) | 0.01144±0.00616 |  |  |
| Never married | 6,081 (14.41%) | 0.01119±0.00567 |  |  |
| **Education level** |  |  | 16.54 | <0.001 |
| High school or below | 20,163 (47.85%) | 0.01209±0.00680 |  |  |
| University or above | 21,974 (52.15%) | 0.01107±0.00586 |  |  |
| **Smoking** |  |  | 348.21 | <0.001 |
| Never | 23,286 (55.21%) | 0.01088±0.00607 |  |  |
| Smoking cessation | 10,272 (24.35%) | 0.01197±0.00665 |  |  |
| Smoking at present | 8,620 (20.44%) | 0.01289±0.00646 |  |  |
| **Drinking** |  |  | 10.62 | <0.001 |
| No | 7,447 (22.16%) | 0.01251±0.00804 |  |  |
| Yes | 26,154 (77.84%) | 0.01145±0.00600 |  |  |
| **Hypertension** |  |  | 16.28 | <0.001 |
| No | 24,419 (57.91%) | 0.01112±0.00568 |  |  |
| Yes | 17,750 (42.09%) | 0.01217±0.00712 |  |  |
| **Diabetes** |  |  | 21.02 | <0.001 |
| No | 35,605 (84.42%) | 0.01125±0.00616 |  |  |
| Yes | 6,573 (15.58%) | 0.01321±0.00707 |  |  |
| **Cardiorenal syndrome** |  |  | 12.75 | <0.001 |
| No | 40,723 (96.55%) | 0.01147±0.00626 |  |  |
| Yes | 1,455 (3.45%) | 0.01418±0.00805 |  |  |
| **Ln MHR** | -4.5756±0.47867 |  |  |  |

* Continuous variables are represented by Mean±SD, while categorical variables are denoted by (n, %).

**Supplementary Table 5.** Baseline characteristics of participants according to BMI

| **Variables*** | **Respondents** | **BMI** | **t/F** | ***P*** |
| --- | --- | --- | --- | --- |
| **Total** | 42,178 | 29.19±6.94 |  |  |
| **Age** | 49.56±17.91 |  |  |  |
| **Gender** |  |  | 13.64 | <0.001 |
| Male | 20,432 (48.44%) | 28.72±6.06 |  |  |
| Female | 21,746 (51.56%) | 29.63±7.64 |  |  |
| **Race** |  |  | 351.07 | <0.001 |
| Mexican American | 6,710 (18.55%) | 29.91±6.18 |  |  |
| Other Hispanic | 3,866 (10.68%) | 29.34±6.21 |  |  |
| Non-Hispanic White | 12,167 (33.62%) | 28.81±6.77 |  |  |
| Non-Hispanic Black | 8,943 (24.73%) | 30.78±8.02 |  |  |
| Other Race | 4,492 (12.42%) | 26.33±5.84 |  |  |
| **Marriage status** |  |  | 54.07 | <0.001 |
| Married/Living with a partner | 25,324 (60.08%) | 29.15±6.53 |  |  |
| Widowed/Divorce/Separated | 10,752 (25.51%) | 29.66±7.31 |  |  |
| Never married | 6,081 (14.41%) | 28.51±7.81 |  |  |
| **Education level** |  |  | 6.313 | <0.001 |
| High school or below | 20,163 (47.85%) | 29.41±6.87 |  |  |
| University or above | 21,974 (52.15%) | 28.99±6.99 |  |  |
| **Smoking** |  |  | 124.97 | <0.001 |
| Never | 23,286 (55.21%) | 29.29±7.06 |  |  |
| Smoking cessation | 10,272 (24.35%) | 29.78±6.61 |  |  |
| Smoking at present | 8,620 (20.44%) | 28.22±6.90 |  |  |
| **Drinking** |  |  | 11.10 | <0.001 |
| No | 7,447 (22.16%) | 30.07±7.11 |  |  |
| Yes | 26,154 (77.84%) | 29.04±6.89 |  |  |
| **Hypertension** |  |  | 41.14 | <0.001 |
| No | 24,419 (57.91%) | 28.00±6.41 |  |  |
| Yes | 17,750 (42.09%) | 30.82±7.29 |  |  |
| **Diabetes** |  |  | 39.56 | <0.001 |
| No | 35,605 (84.42%) | 28.00±6.41 |  |  |
| Yes | 6,573 (15.58%) | 30.82±7.29 |  |  |
| **Cardiorenal syndrome** |  |  | 4.77 | <0.001 |
| No | 40,723 (96.55%) | 28.58±6.65 |  |  |
| Yes | 1,455 (3.45%) | 32.50±7.51 |  |  |

* Continuous variables are represented by Mean±SD, while categorical variables are denoted by (n, %).

**Supplementary Table 6.** Baseline characteristics of participants according to eGFR (ml/min/1.73 m^2^)

| **Variables*** | **eGFR≥60 (n=38,336)** | **45≤eGFR<60 (n=2484)** | **30≤eGFR<45 (n=936)** | **15≤eGFR<30 (n=296)** | **eGFR<15**  **(n=126)** |
| --- | --- | --- | --- | --- | --- |
| **CRS** |  |  |  |  |  |
| No | 38,336 (100%) | 1,663 (66.9%) | 512 (54.7%) | 143 (48.3%) | 69 (54.8%) |
| Yes | 0 (0%) | 821 (33.1%) | 424 (45.3%) | 153 (51.7%) | 57 (45.2%) |
| **Age** | 47.29±16.91 | 71.88±10.08 | 74.37±8.42 | 72.52±11.16 | 61.56±13.98 |
| **Gender** |  |  |  |  |  |
| Male | 18,606 (48.5%) | 1,215 (48.9%) | 417 (44.6%) | 118 (39.9%) | 76 (60.3%) |
| Female | 19,730 (51.5%) | 1,269 (51.1%) | 519 (55.4%) | 178 (60.1%) | 50 (39.7%) |
| **Race** |  |  |  |  |  |
| Mexican American | 6,403 (16.7%) | 187 (7.5%) | 72 (7.7%) | 28 (9.5%) | 20 (15.9%) |
| Other Hispanic | 3,651 (9.5%) | 139 (5.6%) | 51 (5.4%) | 17 (5.7%) | 8 (6.3%) |
| Non-Hispanic White | 15,854 (41.4%) | 1,554 (62.5%) | 576 (61.5%) | 155 (52.3%) | 28 (22.3%) |
| Non-Hispanic Black | 8,168 (21.3%) | 451 (18.2%) | 188 (20.2%) | 78 (26.4%) | 58 (46.0%) |
| Other Race | 4.260 (11.1%) | 153 (6.2%) | 49 (5.2%) | 18 (6.1%) | 12 (9.5%) |
| **Marriage status** |  |  |  |  |  |
| Married/Living with a partner | 23,320 (60.9%) | 1,365 (55.0%) | 443 (47.4%) | 137 (46.3%) | 59 (46.8%) |
| Widowed/Divorce/Separated | 9,079 (23.7%) | 1,022 (41.2%) | 457 (48.9%) | 146 (49.3%) | 48 (38.1%) |
| Never married | 5,920 (15.4%) | 94 (3.8%) | 35 (3.7%) | 13 (4.4%) | 19 (15.1%) |
| **Education level** |  |  |  |  |  |
| High school or below | 17,990 (47.0%) | 1,357 (54.8%) | 559 (59.9%) | 187 (63.6%) | 70 (55.6%) |
| University or above | 20,316 (53.0%) | 1,120 (45.2%) | 375 (40.1%) | 107 (36.4%) | 56 (44.4%) |
| **Smoking** |  |  |  |  |  |
| Never | 21,396 (55.8%) | 1,247 (50.2%) | 443 (47.3%) | 131 (44.3%) | 69 (54.8%) |
| Smoking cessation | 8,706 (22.7%) | 990 (39.9%) | 407 (43.5%) | 129 (43.5%) | 40 (31.7%) |
| Smoking at present | 8,234 (21.5%) | 247 (9.9%) | 86 (9.2%) | 36 (12.2%) | 17 (13.5%) |
| **Drinking** |  |  |  |  |  |
| No | 6,194 (20.2%) | 768 (39.9%) | 323 (46.9%) | 115 (56.7%) | 47 (53.4%) |
| Yes | 24,505 (79.8%) | 1,155 (60.1%) | 365 (53.1%) | 88 (43.3%) | 41 (46.6%) |
| **Hypertension** |  |  |  |  |  |
| No | 23,693 (61.8%) | 560 (22.5%) | 125 (13.4%) | 33 (11.1%) | 8 (6.3%) |
| Yes | 14,634 (38.2%) | 1,924 (77.5%) | 811 (86.6%) | 263 (88.9%) | 118 (93.7%) |
| **Diabetes** |  |  |  |  |  |
| No | 33,122 (86.4%) | 1,708 (68.8%) | 561 (59.9%) | 154 (52.0%) | 60 (47.6%) |
| Yes | 5,214 (13.6%) | 776 (31.2%) | 375 (40.1%) | 142 (48.0%) | 66 (52.4%) |
| **BMI (kg/m^2^)** |  |  |  |  |  |
| <25 | 11,196 (29.2%) | 578 (23.3%) | 212 (22.6%) | 65 (22.0%) | 45 (35.7%) |
| 25-30 | 12,704 (33.1%) | 907 (36.5%) | 318 (34.0%) | 94 (31.8%) | 31 (24.6%) |
| ≥30 | 14,436 (37.7%) | 999 (40.2%) | 406 (43.4%) | 137 (46.2%) | 50 (39.7%) |
| **MHR** | 0.01141±0.0059 | 0.01264±0.0098 | 0.01401±0.0096 | 0.01401±0.0066 | 0.01269±0.0064 |
| **Monocyte count (×10^3^ cells/μl)** | 0.552±0.19 | 0.604±0.29 | 0.641±0.29 | 0.641±0.21 | 0.587±0.22 |
| **HDL-C (mg/dL)** | 53.48±16.22 | 53.19±16.22 | 50.90±15.50 | 50.63±17.33 | 51.18±16.15 |

* Continuous variables are represented by Mean±SD, while categorical variables are denoted by (n, %).

**Supplementary Table 7.** Univariate logistic regression

| **Variables** | **OR** | **95%CI** | ***P*** |
| --- | --- | --- | --- |
| **MHR**  Quartile 1  Quartile 2  Quartile 3  Quartile 4 | 2.271  Ref.  1.404  1.870  2.901 | 1.970-2.618  1.112-1.774  1.488-2.349  2.346-3.586 | <0.001  0.004  <0.001  <0.001 |
| **BMI (kg/m^2^)** | 1.026 | 1.017-1.035 | <0.001 |
| <25 | Ref. |  |  |
| 25-30 | 1.349 | 1.120-1.625 | <0.001 |
| ≥30 | 1.687 | 1.411-2.016 | <0.001 |
| **Age** | 1.469 | 1.140-1.158 | <0.001 |
| **Gender** |  |  |  |
| Male | Ref. |  |  |
| Female | 1.099 | 0.957-1.261 | 0.181 |
| **Race** |  |  |  |
| Mexican American | Ref. |  |  |
| Other Hispanic | 1.522 | 1.062-2.183 | 0.022 |
| Non-Hispanic White | 3.562 | 2.746-4.621 | <0.001 |
| Non-Hispanic Black | 3.419 | 2.589-4.516 | <0.001 |
| Other Race | 1.902 | 1.297-2.791 | <0.001 |
| **Marriage status** |  |  |  |
| Married/Living with a partner | Ref. |  |  |
| Widowed/Divorce/Separated | 2.717 | 2.360-3.128 | <0.001 |
| Never married | 0.308 | 0.207-0.459 | <0.001 |
| **Education level** |  |  |  |
| High school or below | Ref. |  |  |
| University or above | 0.467 | 0.406-0.538 | <0.001 |
| **Smoking** |  |  |  |
| Never | Ref. |  |  |
| Smoking cessation | 2.271 | 1.968-2.621 | <0.001 |
| Smoking at present | 0.789 | 0.608-1.025 | 0.076 |
| **Drinking** |  |  |  |
| No | Ref. |  |  |
| Yes | 0.265 | 0.230-0.305 | <0.001 |
| **Hypertension** |  |  |  |
| No | Ref. |  |  |
| Yes | 11.268 | 9.205-13.793 | <0.001 |
| **Diabetes** |  |  |  |
| No | Ref. |  |  |
| Yes | 5.939 | 5.140-6.861 | <0.001 |

**Supplementary Table 8.** Sensitivity analyses: the association between Ln MHR and CRS

| **Sensitivity analyses** | **OR/RR*** | **95%CI** | ***P*** |
| --- | --- | --- | --- |
| **Without multiple imputation** |  |  |  |
| Overall | 1.772 | 1.455-2.157 | <0.001 |
| Quartile 1 |  | Ref. |  |
| Quartile 2  Quartile 3  Quartile 4  *P*-trend | 1.236  1.525  2.159 | 0.922-1.656  1.132-2.054  1.641-2.840  <0.001 | 0.156  <0.001  <0.001 |
| **Not considering weights** |  |  |  |
| Overall | 1.917 | 1.689-2.177 | <0.001 |
| Quartile 1 |  | Ref. |  |
| Quartile 2  Quartile 3  Quartile 4  *P*-trend | 1.435  1.629  2.424 | 1.182-1.741  1.348-1.969  2.011-2.921  <0.001 | <0.001  <0.001  <0.001 |
| **Poisson regression model** |  |  |  |
| Overall | 1.213 | 1.100-1.338 | <0.001 |
| Quartile 1 |  | Ref. |  |
| Quartile 2  Quartile 3  Quartile 4  *P*-trend | 1.278  1.493  2.009 | 1.025-1.593  1.197-1.862  1.636-2.468  <0.001 | 0.029  <0.001  <0.001 |

* OR for the Logistic regression model and Bayesian logistic regression model; RR for the Poisson regression model

**Supplementary Table 9.** Sensitivity analyses: the association between BMI and CRS

| **Sensitivity analyses** | **OR/RR*** | **95%CI** | ***P*** |
| --- | --- | --- | --- |
| **Without multiple imputation** |  |  |  |
| Overall | 1.039 | 1.023-1.055 | <0.001 |
| <25 kg/m^2^ |  | Ref. |  |
| 25-30 kg/m^2^  ≥30 kg/m^2^ | 1.236  1.525 | 0.922-1.656  1.132-2.054 | 0.156  <0.001 |
| **Not considering weights** |  |  |  |
| Overall | 1.033 | 1.024-1.043 | <0.001 |
| <25 kg/m^2^ |  | Ref. |  |
| 25-30 kg/m^2^  ≥30 kg/m^2^ | 1.071  1.437 | 0.916-1.251  1.228-1.681 | 0.390  <0.001 |
| **Poisson regression model** |  |  |  |
| Overall | 1.033 | 1.022-1.045 | <0.001 |
| <25 kg/m^2^ |  | Ref. |  |
| 25-30 kg/m^2^  ≥30 kg/m^2^ | 1.044  1.394 | 0.878-1.243  1.171-1.660 | 0.624  <0.001 |

* OR for the Logistic regression model and Bayesian logistic regression model; RR for the Poisson regression model

**Supplementary Table 10.** Sensitivity analyses (without multiple imputation): joint associations of MHR and BMI with CRS

| **BMI (kg/m^2^)** | **MHR** | **OR** | **95%CI** | ***P*** |
| --- | --- | --- | --- | --- |
| <25 | Q1 | Ref. |  |  |
| <25 | Q2 | 1.536 | 0.944-2.499 | 0.084 |
| <25 | Q3 | 2.020 | 1.124-3.630 | 0.002^*^ |
| <25 | Q4 | 2.701 | 1.616-4.515 | <0.001^*^ |
| 25-30 kg/m^2^ | Q1 | 1.351 | 0.826-2.208 | 0.231 |
| 25-30 kg/m^2^ | Q2 | 1.748 | 1.054-2.897 | 0.030^*^ |
| 25-30 kg/m^2^ | Q3 | 1.800 | 1.150-2.816 | 0.010^*^ |
| 25-30 kg/m^2^ | Q4 | 2.118 | 1.334-3.363 | 0.001^*^ |
| ≥30 kg/m^2^ | Q1 | 1.786 | 1.011-3.157 | 0.046^*^ |
| ≥30 kg/m^2^ | Q2 | 1.609 | 1.015-2.551 | 0.043^*^ |
| ≥30 kg/m^2^ | Q3 | 2.229 | 1.388-3.580 | <0.001^*^ |
| ≥30 kg/m^2^ | Q4 | 3.494 | 2.302-5.302 | <0.001^*^ |

* *P*<0.05

**Supplementary Table 11.** Sensitivity analyses (not considering weights): joint associations of MHR and BMI with CRS

| **BMI (kg/m^2^)** | **MHR** | **OR** | **95%CI** | ***P*** |
| --- | --- | --- | --- | --- |
| <25 | Q1 | Ref. |  |  |
| <25 | Q2 | 1.585 | 1.114-2.267 | 0.011^*^ |
| <25 | Q3 | 2.122 | 1.497-3.027 | <0.001^*^ |
| <25 | Q4 | 2.585 | 1.809-3.717 | <0.001^*^ |
| 25-30 kg/m^2^ | Q1 | 1.315 | 0.916-1.894 | 0.139 |
| 25-30 kg/m^2^ | Q2 | 1.772 | 1.269-2.496 | 0.001^*^ |
| 25-30 kg/m^2^ | Q3 | 1.922 | 1.395-2.677 | <0.001^*^ |
| 25-30 kg/m^2^ | Q4 | 2.253 | 1.642-3.127 | <0.001^*^ |
| ≥30 kg/m^2^ | Q1 | 1.435 | 0.982-2.097 | 0.061 |
| ≥30 kg/m^2^ | Q2 | 1.908 | 1.367-2.687 | <0.001^*^ |
| ≥30 kg/m^2^ | Q3 | 2.022 | 1.470-2.811 | <0.001^*^ |
| ≥30 kg/m^2^ | Q4 | 3.777 | 2.803-5.163 | <0.001^*^ |

* *P*<0.05

**Supplementary Table 12.** Sensitivity analyses (Poisson regression model): joint associations of MHR and BMI with CRS

| **BMI (kg/m^2^)** | **MHR** | **RR** | **95%CI** | ***P*** |
| --- | --- | --- | --- | --- |
| <25 | Q1 | Ref. |  |  |
| <25 | Q2 | 1.454 | 1.002-2.110 | 0.049^*^ |
| <25 | Q3 | 1.796 | 1.186-2.718 | 0.006^*^ |
| <25 | Q4 | 2.301 | 1.589-3.332 | <0.001^*^ |
| 25-30 kg/m^2^ | Q1 | 1.180 | 0.798-1.743 | 0.406 |
| 25-30 kg/m^2^ | Q2 | 1.639 | 1.120-2.398 | 0.011^*^ |
| 25-30 kg/m^2^ | Q3 | 1.680 | 1.192-2.368 | 0.003^*^ |
| 25-30 kg/m^2^ | Q4 | 1.956 | 1.379-2.775 | <0.001^*^ |
| ≥30 kg/m^2^ | Q1 | 1.621 | 1.057-2.487 | 0.027^*^ |
| ≥30 kg/m^2^ | Q2 | 1.590 | 1.108-2.280 | 0.012^*^ |
| ≥30 kg/m^2^ | Q3 | 1.996 | 1.391-2.863 | <0.001^*^ |
| ≥30 kg/m^2^ | Q4 | 2.935 | 2.129-4.046 | <0.001^*^ |

* *P*<0.05

**Supplementary Figure 1.** Histograms of MHR and Ln MHR


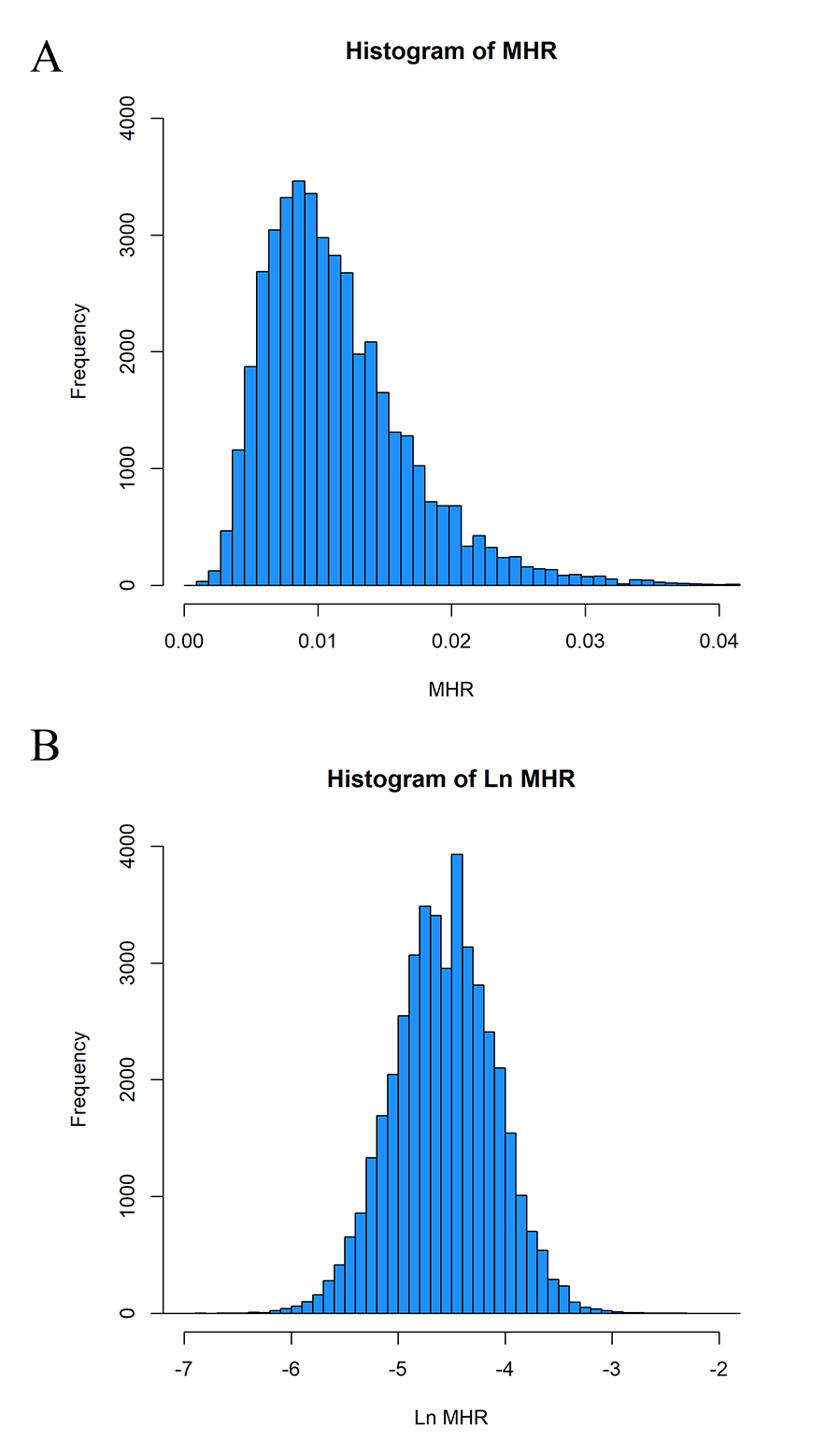


**A.** Distribution of MHR; **B.** Distribution of Ln MHR. **Abbreviation:** the natural logarithmic transformation of MHR.
